# PP21 Abdominal Aortic Aneurysm Screening In Men: Systematic Review Of Cost Effectiveness

**DOI:** 10.1017/S0266462325102419

**Published:** 2025-12-29

**Authors:** Elaine Foley, Karen Jordan, Andrés López, Shelley O’Neill, Máirín Ryan, Susan Spillane

## Abstract

**Introduction:**

Abdominal aortic aneurysm (AAA) is an abnormal dilation of the abdominal aorta, often asymptomatic but potentially fatal if ruptured. Introducing population-based ultrasound screening could support earlier detection and surgical repair. This systematic review assessed the cost effectiveness of population-based ultrasound screening for AAA in men versus no screening, and explored factors influencing cost effectiveness, as part of a health technology assessment.

**Methods:**

Literature searches were conducted in electronic databases and gray literature sources (through to 23 July 2024). Screening, data extraction, quality appraisal (Consensus Health Economic Criteria list) and assessment of transferability (Professional Society for Health Economics and Outcomes Research questionnaire) were performed in duplicate (outlined in protocol CRD42024501141). Findings were presented with reference to the local decision context (Ireland). To facilitate comparison, incremental cost-effectiveness ratios (ICERs) were converted to 2023 EUR. Cost effectiveness was interpreted using willingness-to-pay (WTP) thresholds of EUR20,000 and EUR45,000 per quality-adjusted life year (QALY).

**Results:**

Of 235 reviewed publications, 20 relevant studies were identified for inclusion and analyzed in detail (17 cost-utility analyses [CUAs] and three cost-effectiveness analyses [CEAs]). Screening was found to be cost effective at a WTP threshold of EUR45,000 per QALY in 16 of 17 CUAs (ICERs: EUR200 to EUR30,600 per QALY). CEAs reported ICERs between EUR7,500 and EUR16,100 per life year gained. In sensitivity analyses, AAA screening became less cost effective with decreasing AAA prevalence. None of the studies were considered transferable to the Irish context.

**Conclusions:**

International evidence suggested that AAA screening in men was cost effective. However, despite the consistency of findings in the evidence base, declining AAA prevalence contributed to uncertainty about the long-term cost effectiveness of AAA screening in men.

